# Associating resistance to immune checkpoint inhibitors with immunological escape in colorectal cancer

**DOI:** 10.3389/fonc.2022.987302

**Published:** 2022-09-30

**Authors:** Yi Ding, Zehua Wang, Fengmei Zhou, Chen Chen, Yanru Qin

**Affiliations:** ^1^ Department of Oncology, First Affiliated Hospital of Zhengzhou University, Zhengzhou, China; ^2^ Academy of Medical Sciences, Zhengzhou University, Zhengzhou, China

**Keywords:** ICI, resistance, colorectal cancer, immune escape, overcome resistance

## Abstract

Colorectal cancer is a common malignant tumor that ranks third in incidence and second in mortality worldwide, and surgery in conjunction with chemotherapy and radiotherapy remains the most common treatment option. As a result of radiotherapy’s severe side effects and dismal survival rates, it is anticipated that more alternatives may emerge. Immunotherapy, a breakthrough treatment, has made significant strides in colorectal cancer over the past few years, overcoming specialized therapy, which has more selectivity and a higher survival prognosis than chemoradiotherapy. Among these, immune checkpoint inhibitor therapy has emerged as the primary immunotherapy for colorectal cancer nowadays. Nonetheless, as the use of immune checkpoint inhibitor has expanded, resistance has arisen inevitably. Immune escape is the primary cause of non-response and resistance to immune checkpoint inhibitors. That is the development of primary and secondary drug resistance. In this article, we cover the immune therapy-related colorectal cancer staging, the specific immune checkpoint inhibitors treatment mechanism, and the tumor microenvironment and immune escape routes of immunosuppressive cells that may be associated with immune checkpoint inhibitors resistance reversal. The objective is to provide better therapeutic concepts for clinical results and to increase the number of individuals who can benefit from colorectal cancer immunotherapy.

## Introduction

According to the most recent worldwide statistics, the global cancer burden is overwhelming and expanding, with WHO estimates predicting a global cancer burden of 28.4 million cases in 2040. Colorectal cancer (CRC) ranks third in global cancer incidence while second in global cancer death, and colorectal cancer prevention, diagnosis, as well as treatment, are still significant issues that need to be tackled (1). The Chinese Society of Clinical Oncology (CSCO) colorectal cancer recommendations mainly propose surgery, chemotherapy, and radiation, with the combination of these modalities depending on the location, size, grade, and metastasis of colon cancer (2). Tumor cells are not eradicated even with comprehensive therapy, and the outlook for CRC is not exceptional. Furthermore, traditional therapies are indeed imprecise, causing patients to suffer and have a lower quality of life (3–5).

In recent years, immunotherapy has made tremendous progress in solid malignancies such as melanoma and lung cancer (6). Immunotherapy enhances the immune response against cancer cells by improving detection of tumor cell antigens (7, 8). Among them, immune checkpoint inhibitors (ICIs) have achieved the most significant improvements in immunotherapy, achieving rates of durable remission that are unprecedented. The majority of patients, however, have not benefited from treatment, and some in remission have relapsed after a period of remission because they have established medication resistance.

Typically, drug resistance is classified as either primary or secondary. The heterogeneity of the tumor growth process may play a role in the mechanism of primary and secondary ICIs resistance. There is no clear and comprehensive explanation of the immunological medication resistance mechanism. However, regardless of the resistance pattern, immunological escape is the underlying phenomenon. ICIs in CRC are essentially targeted to the MSI-H staging. pMMR is more like a “cold tumor” in the treatment of ICIs and has no more treatment options. In this article, we discuss the immune treatment-related CRC staging, the specific modalities of ICIs treatment, and the tumor microenvironment (TME) and immune escape pathways of immunosuppressive cells that may be associated with the reversal of ICIs resistance. Furthermore, we explore not only MSI-H-related ICIs therapy but also the role of immune escape to inspire the future of inhibiting certain TME or immunosuppressive cells to convert pMMR to “hot tumors” in order for ICIs to be effective. The goal is to obtain more beneficial therapeutic ideas for clinical outcomes and to expand the population benefiting from CRC immunotherapy.

## CRC mutation pattern typing

The immune system eliminates highly immunogenic tumor cells in the body. Tumor cells continuously undergo somatic mutations and passively select low-immunogenic variants for proliferation in response to screening for antitumor effects. The MMR/MSI system classification has been significant in the treatment of colorectal cancer.16% of CRCs are hypermutated, of which 75% are connected to MSI-H and 25% have mutations in somatic mismatch repair genes and polymerase E (pole) (9). As a complex enzyme proofreading system, MMR is active during DNA replication, correcting nucleotide pairing mismatches and sliding between the two strands of DNA. Nevertheless, an insertion or deletion mutation during DNA replication cannot be corrected if the MMR mechanism is flawed. A shortened non-functional protein fragment, MSI, is eventually formed as a result of a germ-line mutation in one of the MMR system genes (MLH1, MSH2, MSH6, PMS2, or TACSTD1/EpCAM), or hypermethylation of the MLH1 promoter (10–12). The genome is full of microsatellite sequences, which are polymorphic between individuals yet specific to each tissue of each person.

In order to maintain genomic stability, mismatch repair (MMR) correctly identifies and corrects base mismatches, small base deletions, and insertions that arise during DNA replication or recombination. MMR is subdivided into different mismatch repair (dMMR) and proficient mismatch repair (pMMR). pMMR refers to normal MMR expression. dMMR refers to mutations in genes involved in MMR repair that lead to impairments in gene function and reduced or missing MMR repair competence. MSI is a code-shifting mutation caused by the insertion or deletion of repeating units in tumor cells’ microsatellites. pMMR manifests as low-frequency microsatellite instability (MSI-L) or microsatellite stability (MS-S), whereas dMMR manifests as high-frequency microsatellite instability (MSI-H) (13–17). MSI is a crucial component in the progression of CRC (18). The principal method by which CRC can merge with MSI is through the methylation and inactivation of the hMLH1 promoter, which results in mismatch repair mistakes in certified identity management professionals (19), microsatellite sequences are abundant throughout the entire genome; they are polymorphic between individuals but unique in each tissue of each individual (20).

## Existing mainstream immunotherapy for CRC

**Figure 1:**
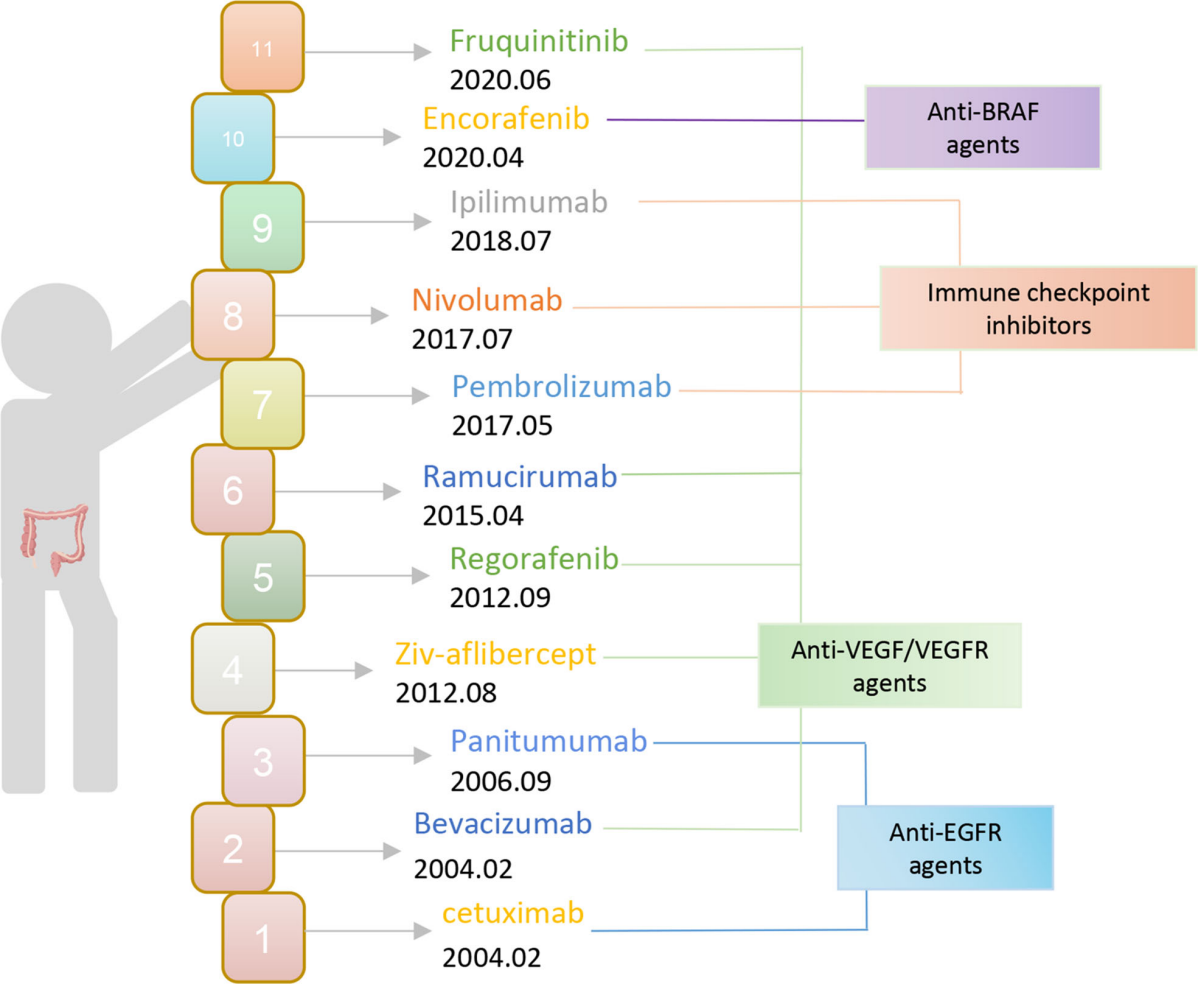
The FDA approves immunotherapy for CRC.

Currently, the chosen clinical treatment technique for colorectal cancer is still predominantly surgery and radiotherapy, despite the unsatisfactory overall effect. To put it another way, immunotherapy has advanced quickly in the field of oncology in recent years and has achieved excellent results in the treatment of solid tumors like melanoma and lung cancer. Therefore, offering novel concepts for the treatment of colorectal cancer and making immunotherapy a well-liked research topic in CRC treatment. The FDA authorized bevacizumab and cetuximab for use as first-line CRC medications in 2004 (**Figure 1**). Monoclonal antibodies have since entered the CRC immunotherapy arena. The inhibitors of vascular endothelial growth factor (VEGF) and epidermal growth factor receptor (EGFR) still play an important role as a therapeutic aid today. Then the FDA approved pembrolizumab, an ICI PD-1 monoclonal antibody, in 2017 for the treatment of dMMR-MSI-H. With ICI, immunotherapy is now making the progress in colorectal cancer. Subsequently, the FDA expedited approval of the anti-PD-1 monoclonal antibodies pembrolizumab and nivolumab, in addition to nivolumab and the CTLA-4 monoclonal antibody ipilimumab for the treatment of dMMR-MSI-H. However, ICIs are ineffective against pMMR-MSI-L colorectal cancer almost. Yet patients with dMMR-MSI-H account for less than 5% of all colorectal cancer patients (21). It has been assumed that immunological tolerance and immunotherapy are ineffective in pMMR-MSI-L CRC due to the low number of mutations and the minimal penetration of immune cells (22, 23).

In addition to ICI, the FDA has approved chimeric antigen receptor T-cell (CAR-T) therapy for clinical use. Through attaching the B-cell receptor antigen-binding domain to the T-cell receptor’s intracellular area, which is subsequently genetically altered for return to the body, this therapy employs genetically modified immune cells to express autologous T cells that can recognize and destroy tumor cells (24, 25). CAR-T cells are currently restricted to clinical applications targeting the pan-B cell antigen CD19 and are only licensed for the treatment of certain hematological cancers. A significantly higher fraction of activated cytotoxic CD8 TIL in colorectal cancer patients indicated that the degree of T cell penetration into the tumor was directly related to treatment success and suggested that immunological editing could inhibit tumor growth (26). T cells react strongly to antigens from infections but are insensitive to antigens from cells that are unable to exert a comparable cell-killing impact *in vivo*. CAR-T therapy collects T cells from tumors, peripheral blood, or lymph nodes of patients for genetic engineering. Not only because the use of autologous T cells can reduce immune rejection, but also because the use of autologous T cells can promote the identification of tumor cells and increase T cell activation (27). While CAR-T therapy has not yet been approved for the clinical treatment of colorectal cancer, no serious adverse events associated with CAR-T therapy were observed in the 2017 Zhang et al. study (28). CAR-T therapy in patients with carcinoembryonic antigen (CEA)-positive colorectal cancer (CRC) metastases is effective, with 7 out of 10 patients experiencing stable disease within four weeks of CAR-T cell infusion and another 2 patients experiencing tumor shrinkage with the treatment being well-tolerance (28).Aside from the experimental CEA target, there are numerous potential targets for CAR-T cell therapy in colorectal cancer, primarily anti-4-1BB (ANTI-4-1BB), CEA, Guanylate Cyclase 2C(GUCY2C), Tumor Associated Glycoprotein 72(TAG-72), Epithelial cell adhesion molecule(EpCAM), epithelial glycoprotein 40 (EGP40), NKG2D, human epithelial growth factor receptor-2(HER-2), recombinant human interferon-alpha/beta receptor 1 (IFNAR1), prominin-1 (CD133), epithelial glycoprotein 2 (EGP-2), etc. Active research is also being conducted on the clinical translation of non-traditional immune cells other than T cells in colorectal cancer immunotherapy.

Vaccines are the oldest treatment connected with immunity, and in recent years, tumor vaccine research has been one of the most active fields of study. In 2019, A.E. Snook et al. conducted a phase I clinical trial evaluating the tumor vaccine Ad5-GUCY2C-PADRE(adenovirus vector vaccine) in patients with early-stage colorectal cancer, none of which had an adverse event greater than grade I. GUSY2C antibody responses were seen in 10% of patients, while GUSY2C-specific CD8 cytotoxic T cell responses were seen in 40% of patients (29). Furthermore, the PolyPEPI1018 vaccination (peptide vaccine for conserved cancer antigen expression) combined with first-line maintenance therapy may be the treatment of the future for MSS CRC, for which immunotherapy is rather inefficient. In 11 patients with MSS-mCRC, Joleen M. Hubbard et al. administered subcutaneous injections of PolyPEPI1018 with first-line treatments. Ultimately, there were no serious side effects from the vaccine, five patients got a single dose of PolyPEPI1018 and six patients got up to three doses every 12 weeks. Three patients progressed, five patients were stable, and three patients had partial tumor remission, two of whom had tumors that were small enough to undergo surgery (30). This is an advancement for MSS-CRC.

Bispecific antibodies, a novel immunotherapy concept, are already under investigation for the treatment of CRC. Apart from that, bispecific antibodies, as opposed to monoclonal antibodies, can simultaneously bind to two antigenic epitopes. Bacac et al. created CEA-TCB as a bispecific antibody that binds both CEA expression on cancer cells and CD3 on T cells. This way leads directly to the binding of T cells to cancer cells without the intervention of other immune systems, thereby inducing direct autoimmune destruction of tumor cells (31). As with CRC, which typically has high CEA expression, the progression of CEA-TCB is expected.

In this sense, immunotherapy is more innovative than conventional approaches in that it can produce unique therapeutic results. Nonetheless, the immune system is a part of the individual’s system, and excessive interference might result in adverse side effects. ICI, a large class of immunosuppressive pathways present in the body, such as regulate T- cell responses by blocking immune checkpoints (ICs) and increasing T-cell activation. However, over-activation may result in systemic autoimmune disorders (32, 33). At the same time, the widespread use of ICI has led to the development of acquired resistance in many patients who initially responded favorably. The relatively poor scientific and technical understanding of the mechanisms of acquired ICI resistance may hinder the development of immunotherapies for the next generation (**Table 1**).

**Table 1:** Ongoing clinical trials of immunotherapy for CRC patients.

NCT number	Study Title	Phase	Strategy	Primary outcomemeasures	genomic stratification
NCT03206073	Pexa-Vec Oncolytic Virus With Immune Checkpoint Inhibition	I/II	PD-L1,CTLA4 Inhibitors and Oncolytic virus	Rate of AEs	MSS
NCT03388190	Anti-tumor IMMunity by OXaliplatin	II	PD-1 Inhibitors and Chemotherapy	PFS	MSS/pMMR
NCT03287427	MYB and PD-1 Immunotherapies	I	Vaccine and PD-1 Inhibitors	Rate of AEs and DLTs	N/A
NCT01885702	Dendritic Cell VacciN/Ation	I/II	Vaccine	Safety and feasibility of vacciN/Ation	MSI-H
NCT04044430	Encorafenib, Binimetinib, and Nivolumab	I/II	MEK,BRAF and PD-1 Inhibitors	Radiographic Response	MSS
NCT03435107	Durvalumab	II	PD-L1 Inhibitors	ORR	POLEmutated/MSI-H
NCT02437071	Pembrolizumab Plus Radiotherapy or Ablation	II	PD-1 Inhibitors,Radiotherapy and RFA	response rate	N/A
NCT02754856	Tremelimumab and Durvalumab	I	PD-L1,CTLA4 Inhibitors,Laboratory Biomarker AN/Alysis and Surgery	Feasibility	N/A
NCT02983578	Danvatirsen and Durvalumab	II	STAT and PD-L1 Inhibitors	Rate of AEs, SAEs	MMR
NCT03800602	Nivolumab and Metformin	II	Metformin and PD-1 Inhibitors	ORR	MSS
NCT03851614	Inhibitors of DN/A Damage Response, Angiogenesis and Programmed Death Ligand 1	II	PD-L1, PARP and VEGFR2 Inhibitors	Changes in genomic and immune biomarkers	MMR
NCT03639714	PersoN/Alized Neoantigen Cancer Vaccine	I/II	Vaccine	Rate of AEs, SAEs, DLTs	MSS
NCT03436563	M7824	I/II	PD-1 Inhibitors and TGFbetaRII Fusion Protein	ORR	MSI
NCT02873195	Capecitabine and Bevacizumab With or Without Atezolizumab i	II	PD-L1,VEGF Inhibitors and Chemotherapy	PFS	N/A
NCT03290937	Utomilumab, Cetuximab, and Irinotecan Hydrochloride	I	EGER,4-1BB Inhibitors and Chemotherapy	Recommended phase 2 dose of irinotecan hydrochloride	N/A
NCT03228667	CombiN/Ation Immunotherapies	II	N-803,PD-1and PD-L1 Inhibitors	ORR	MSI-H
NCT03186326	Chemotherapy vs Immunotherapie	II	PD-L1, VEGFR, VEGF and EGFR Inhibitors	PFS	MSI
NCT02903914	ArgiN/Ase Inhibitor INCB001158 With Immune Checkpoint Therapy	I/II	PD-1 and ARG I Inhibitors	Rate of AEs	N/A
NCT03610490	MDA-TIL	II	MDA-TIL and Chemotherapy	ORR	N/A
NCT04721301	Ipilimumab, Maraviroc and Nivolumab	I	PD-1,CTLA-4 and CCR5 Inhibitors	Rate of AEs	N/A
NCT03981146	Nivolumab	II	PD-1 Inhibitors	Durable Clinical Benefit	MSS
NCT02888743	Durvalumab and Tremelimumab With or Without Radiation Therapy	II	PD-1,CTLA-4 Inhibitors and Radiation Therapy	ORR	MSS
NCT03712943	Regorafenib and Nivolumab	I	PD-1 and VEGFR Inhibitors	Maximum Tolerated Dose	MMR
NCT03547999	Perioperative CV301 VacciN/Ation With Nivolumab and Systemic Chemotherapy	II	PD-1 Inhibitors, Vaccine and Chemotherapy	OS	N/A
NCT03174405	Avelumab and Cetuximab With FOLFOX in Patients The Phase II AVETUX-CRC	II	PD-L1 Inhibitors	PFS	MSI/MSS
NCT03396926	Pembrolizumab, Capecitabine, and Bevacizumab	II	PD-1 and VEGF Inhibitors,and Chemotherapy	Frequency of treatment-related DLT	MSS
NCT03658772	Grapiprant and Pembrolizumab	I	PD-1 and EP4R Inhibitors	Safety and tolerability of grapiprant alone	MSS
NCT02740985	AZD4635	I	PD-L1, A2AR Inhibitors and Chemotherapy	DLTs	MSS
NCT03867799	iSCORE : Immunotherapy Sequencing	II	PD-1 Inhibitors	DCR	N/A
NCT03289962	Autogene Cevumeran (RO7198457) With Atezolizumab	I	PD-L1 Inhibitors and Vaccine	DLTs	N/A
NCT04208958	VE800 and Nivolumab	I/II	PD-1 Inhibitors, Antibiotics and Microbial Therapy	Safety and tolerability	MSS
NCT03948763	mRN/A-5671/V941 With Pembrolizumab (V941-001)	I	PD-1 Inhibitors and Vaccine	DLTs and rate of AEs	non-MSI-H
NCT03350126	Nivolumab and Ipilimumab	II	PD-1, CTLA-4 Inhibitors	DCR,PFS,ORR	MSI/MMR
NCT03507699	Immunotherapy and Radiosurgery	I	PD-1,CTLA-4 Inhibitors and TLR9 agonist	DLTs	non-MSI-H
NCT01787500	Vemurafenib, Cetuximab, and Irinotecan Hydrochloride	I	BRAF Inhibitors and Chemotherapy	DLTs	N/A
NCT04513951	AVELUMAB and CETUXIMAB and mFOLFOXIRI	II	PD-1, EGFR Inhibitors and Chemotherapy	Rate of ORR,PFS and Toxicity	N/A
NCT03414983	Nivolumab With Standard of Care Therapy *vs* Standard of Care Therapy	II/III	PD-1 Inhibitors and Chemotherapy	Rate of ORR and PFS	N/A
NCT03170960	Cabozantinib With Atezolizumab	I/II	Tyrosine kiN/Ase and PD-L1 inhibitor	Rate of MTD, ORR and PFS	N/A
NCT03721653	FOLFOXIRI + Bev + Atezo *vs* FOLFOXIRI + Bev	II	PD-1, VEGF Inhibitors and Chemotherapy	Rate of ORR,PFS and Toxicity	N/A
NCT02060188	Nivolumab Alone or Nivolumab CombiN/Ation Therapy	II	PD-1,CTLA-4 and MEK Inhibitors and Anti-Human CD38	Rate of ORR	MSI
NCT03849469	XmAb^®^22841 Monotherapy With or Without Pembrolizumab	I	XmAb^®^22841 and PD-1 Inhibitors	Rate of AEs	N/A
NCT03373188	VX15/2503 and Immunotherapy	I	Anti-SEMA4D PD-1,CTLA-4 Inhibitors and Surgery	Evaluate treatment effects,rate of AEs	MSS
NCT02009449	Pegilodecakin (LY3500518)	I	PD-1,VEGF Inhibitors and Chemotherapy	Rate of AEs	N/A
NCT03761914	Galinpepimut-S With Pembrolizumab	I/II	PD-1 Inhibitors and Vaccines	Rate of ORR, CR and TRAEs	N/A
NCT03184870	BMS-813160 With Chemotherapy or Nivolumab	I/II	PD-1 Inhibitors and Chemotherapy	Rate of AEs and DLT’S	N/A
NCT03095781	Pembrolizumab and XL888	I	PD-1 and HSP90 Inhibitors	Rate of AEs and OS	N/A
NCT03239145	Pembrolizumab (Anti-PD-1) and AMG386 (Angiopoietin-2 (Ang-2)	I	PD-1 and VEGF Inhibitors	Rate of ORR,OS,PFS and DLT’S	N/A
NCT04306900	TTX-030 With Immunotherapy With or Without Chemotherapy	I	TTX-030 and PD-1 Inhibitors	Rate of AEs	N/A

N/A, not applicable.

## Exploration of ICIs’ antitumor effects and resistance mechanisms

### Immunosuppressive cells

#### TREG

Regulatory T cells (Treg) are present in both the thymus and the periphery, with natural Treg in the thymus promoting autoimmune tolerance and degenerating with age, whereas peripherally adaptive Treg are antigen-specific suppressor cells that can be converted to Treg by CD4+CD25+ T cells induced by tumor cells, thereby promoting immune escape (34, 35). By lowering the immune response in healthy humans, Tregs can avoid autoimmune disorders. Through a cytokine-dependent or cell-cell contact mechanism, however, Tregs in cancer patients inhibit the immune response to the tumor (36).

Gershon R.K. introduced the concept of immunosuppressive T cells in 1974, demonstrating the crucial role of such T cells in both *in vivo* and *ex vivo* suppressive effects (37). Treg’s existence has been demonstrated in recent years by research demonstrating its ability to block tumor rejection (38–41). In 1999, Onizuka, S and Shimizu, J investigated the role of Treg in tumor immunity in mice for the first time. Subsequent studies demonstrated that Treg cells have a negative effect on CTL production as well as the innate immune response, and animal experiments demonstrated that a decrease in the number of Treg cells correlates with a decrease in tumor size. There is a link between a decrease in the number of Treg cells and a diminution in tumor volume (42–45). Besides that, Somasundaram, R. et al. investigated the utility of Treg in CRC in 2002 and discovered that Treg is induced by TGF-β in human colorectal cancer without contact and mediates immune escape to protect tumor cells by inhibiting CTL activation and subsequently acting as a mechanism to inhibit tumor cell destruction (46). The statistical analysis of the case studies revealed that elevated Treg was associated with a poor prognosis for CRC and with recurrent metastases following CRC tumor excision (47). Tumor cells drive the aggregation and synthesis of Treg through a variety of mechanisms throughout tumor progression. In cancer, the release of chemokines CCL17, CCL22, and CCL28 stimulates Treg recruitment (48–50). Autoimmunity exerts an anti-tumor cell effect during the early stages of tumor growth by detecting tumor cell autoantigens and rejecting them. However, as the tumor process advances, CTL-mediated autoimmunity is finally defeated by immunosuppression established through Treg cells. When the number of Treg cells surpasses the number of effector T cells, immunological escape is encouraged (38, 39, 51). Through comparison of a tumor-bearing mouse model constructed by Barbara Valzasina et al. with a tumor-free mouse model, it was identified that Treg promotes immune escape by suppressing the proliferation of existing T cells that continuously interact with dendritic cells to maintain the effects of providing autoantigen and costimulation; in addition to favoring the generation of a broader T cell lineage from the circulation to promote immune escape with new Treg (34). The work by Ngiow S F et al. indicated that intra-tumor Tregs are partly responsible for the formation of anti-PD1 resistant tumors and PD1(hi)CD8(+) T cells. Furthermore, the reduction in the CD8+T/Treg ratio can be used to demonstrate the efficacy of an anti-PD-1 monoclonal antibody (52).

#### MDSCs

After the 1980s, through extensive research on tumor patients, suppressor myeloid cells were identified and characterized. These myeloid cells with suppressive activity were later collectively referred to as myeloid-derived suppressor cells (MDSCs), and there is now abundant evidence that MDSCs play a suppressive role in the immune system. They share a myeloid origin, an immature condition, and a remarkable capacity to inhibit T-cell responses (53–55). MDSCs originate from myeloid cells that failed to differentiate and mature as a result of cancer, inflammation, trauma, autoimmune disorders, etc. Myeloid progenitor cells and immature myeloid cells comprise this diverse cell type (IMCs) (56). The primary manifestation of MDSC immunosuppression is the inhibition of T cell proliferation and the promotion of Treg formation. There is evidence that elevated circulating levels of MDSCs correlate with disease stage, classification, and metastasis development in advanced colorectal cancer (57). MDSCs regulate the metabolism of L-arginine *via* inducible nitric oxide synthase (INOS) and arginase-1 (ARG1), which depletes the microenvironment of L-arginine, inhibits T cell proliferation, cytokine production, and expression of the T cell receptor CD3 zeta chain, converts L-arginine into polyamines, and promotes tumor growth. L-arginine is induced by INOS to create NO and ROS, which lowers CD3 zeta expression and triggers T-cell death (58–63). The synthesis of arginase II by mature myeloid cells such as macrophages does not drain L-Arg from the microenvironment and does not compromise the function of T-cells (64). In 1993, Nakagomi H discovered that T cells isolated from colorectal cancer patients expressed much less CD3 zeta than peripheral blood T cells from the same patients, and that peripheral blood zeta chain levels were significantly lower than T cell zeta chain levels in lymphocytes (65). Ichihara et al. examined the expression of CD3 zeta in peripheral blood mononuclear cells before and after surgery in 28 patients and found that hydrogen peroxide-mediated stimulation of mononuclear cells decreased the expression of TCR CD3 zeta molecules in peripheral T cells (66). Mizoguchi H had previously hypothesized that T cells from mice with tumors exhibit T cell antigen receptors with little CD3γ and no CD3 zeta, which are substituted by Fc epsilon γ chains. Also diminished was the expression of the tyrosine kinases p56lck and p59fyn. These modifications may be the cause of immunodeficiency in the tumor-bearing host (67). In conclusion, the study of MDSCs is still in its infancy and a great deal of research is still being conducted, but it is already known from current experiments that the level of MDSCs in clinical patients is closely related to the efficacy of immunotherapy and the prognosis of patients. Patients with colorectal cancer who have a poor response to conventional immunotherapy pMMR-MSI-L staging may, potentially, benefit considerably from immunotherapies that specifically target MDSCs. Additionally, the absence of ARG1 activity can reduce the effectiveness with which MDSCs can be inhibited and enhance the sensitivity of PD-1/PD-L1 antibodies (68).

#### TAMS

Pelka et al. used single-cell RNA sequencing and spatial analysis to compare a large number of colorectal cancer patients’ tissues with normal tissues, which had more monocytes and macrophages than normal tissues (69). After specific differentiation, macrophages can be divided into two different polarization states based on their function and level of inflammatory factor secretion: M1 and M2-macrophages (70–73). M1 macrophages boost the Th1 response by ingesting and destroying the target tumor cells. M2 macrophages release anti-inflammatory cytokines that promote angiogenesis and the beginning and progression of tumors (73, 74). In addition, when tumor cells are present, immune cells might connect to them and develop a unique biological phenotype as a result of their interaction. M-MDSCs may then develop into tumor-associated macrophages (TAMs), have an M2-like phenotype, and enhance anti-tumor immunosuppression by promoting tumor angiogenesis or indirectly interfering with the interactions of immune cells in the tumor microenvironment (TME). In the interim, TAMs can attract Tregs by secreting chemokines, allowing Tregs to inhibit T cells through anti-tumor immunological responses (75–78). TAMs are intrinsically inhibitory of CTL cell function, blocking TCR signaling while increasing T cell unresponsiveness and death *via* increased expression of PD-L1 and CTLA-4 in association with the relevant receptors on CTL cells (79). TAMs can also promote tumor development, invasion, metastasis, immunosuppression, angiogenesis, and drug tolerance by secreting cytokines and chemokines that coordinate with inflammatory mechanisms, as demonstrated by the TGF-β,VEGF, PDGF, M-CSF, IL-10, and CXCL (80). In a mouse model of pancreatic cancer already proven, however, inhibiting macrophage CSF-1R (colony-stimulating factor 1 receptor), reducing the frequency of TAM, and increasing IFN production can increase the responsiveness of tumor cells to the treatment. Gemcitabine was much more effective when combined with CSF-1R blockers and PD-1 or CTLA-4 antibodies. It will be worthwhile to wait for equivalent colorectal cancer evidence (81).

### The role of immune checkpoints in the treatment of CRC by the mechanism

Immune checkpoint molecules, such as PD-1, PD-L1, and CTLA4, can activate signaling pathways that restrict T-cell function. They are a class of immunosuppressive molecules that are expressed on immune cells to control the level of immune activation. And this type is currently the most frequently targeted immunotherapy agent. James Allison of the United States and Tasuku Honjo of Japan were awarded the 2018 Nobel Prize in Physiology or Medicine for their contributions to the discovery of negative immune regulation, also known as CTLA4 and PD-1, as cancer treatments (82).

**Figure 2:**
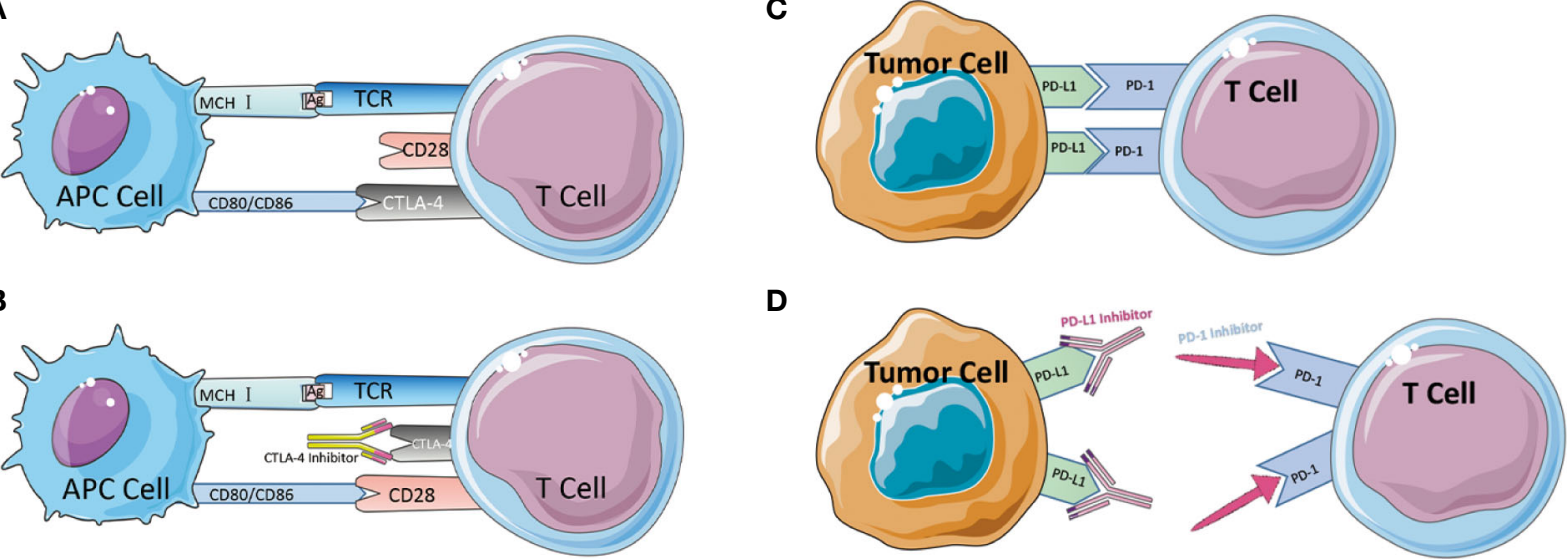
**(A)** Schematic diagram of CTLA-4-mediated immune escape. **(B)** Schematic diagram of CTLA-4 inhibitor to suppress immune escape. **(C)** Schematic diagram of PD-1/PD-L1-mediated immune escape. **(D)** Schematic diagram of PD-1/PD-L1 inhibitor to suppress immune escape.

CTLA-4 is a protein receptor that inhibits the immunological response in humans. CTLA-4 is expressed on the surface of CD4+ and CD8+ lymphocytes. It competes with the T-cell costimulatory receptor CD28 for interactions with T-cell costimulatory factors and, by binding to CD28 (**Figure 2**), reduces T-cell proliferation (83–85). These pathways maintain autoimmune tolerance and regulate the duration and magnitude of physiological immune responses induced by peripheral tissues. ICs physiologically prevent autoimmunity by inhibiting immune cells’ responses. It is typically initiated by ligand-receptor interactions and can be inhibited by antibodies or by recombinant forms of ligands or receptors (84, 86, 87).Despite this, these molecules are frequently chosen as the primary immune evasion mechanism following the development of tumors. When the FDA approved the CTLA-4 monoclonal antibody Ipilimumab as an immunotherapy for metastatic melanoma in 2011, it was the first time an ICI had been approved for clinical use as a cancer immunotherapy medicine. However, the therapeutic treatment of CRC with the CTLA-4 monoclonal antibody did not demonstrate the anticipated efficacy. Chung conducted a single-arm, multi-center phase II intravenous monoclonal antibody trial utilizing Tremelimumab on 47 patients, with only one patient obtaining a second therapy and reaching a six-month partial remission. The overall survival (OS) median was 19.1 months and the progression-free survival (PFS) median was 2.3 months (88). The trial did not utilize MSI-H in regard to the MMR subgroup, but the results of this trial imply that CTLA-4 monoclonal antibody may not be suitable for CRC monotherapy.

Besides CTLA-4, the Programmed Death 1 receptor and its ligand (PD-1/PD-L1) are an IC that was discovered in 1992 by Tasuku Honjo in a mouse T-cell hybrid tumor (89). PD-1 is an inhibitory co-receptor expressed on NK cells, B cells, T cells, and TIL cells, indicating that PD-1 has a broader function than CTLA-1. The team of L CHEN released in 1999 an article describing the discovery that the B7-H1 molecule (PD-L1), which can bind to PD-1, co-regulates certain cellular immune responses. In healthy organisms, the interaction between PD-1/PD-L1 restricts T cell effector responses in order to maintain immunological dynamic equilibrium and protect the body against autoimmunity with severe inflammation. PD-L1 is expressed on activated lymphocytes (T cells, B cells, and NK cells), peripheral tissues, and organs. After binding, PD-1 inhibits the kinase that activates T cells *via* the phosphatase SHP250. PD-1 can also inhibit TCR signaling, thereby altering the duration of T cell-APC or T cell-target cell contacts. The combination of PD-1 and PD-L1 induces apoptosis, depletion, and hypofunction in T cells, which in turn inhibits the activation, proliferation, and antitumor activity of CD8+ T cells specific to tumor antigen (79, 89–94). Tumor tissue regulates enhanced PD-1 expression, permitting more PD-1 to bind to ligands, inhibit cytotoxic cells, and limit the release of related cytokines (95). In the absence of a matching therapeutic application, PD-1 can also attach to PD-L2, which is associated with the inhibition of immunological responses and immune tolerance. In 2014, the FDA approved two PD-1 monoclonal antibodies, Nivolumab and Pembrolizumab, for the clinical treatment of metastatic melanoma (**Figure 2**). In 2010, Julie R. Brahmer completed a single-agent Phase I clinical trial with PD-1 monoclonal antibody involving a total of 14 patients with CRC, one of whom achieved lasting full remission. A subsequent phase II clinical trial that added dMMR status to the evaluation criteria ultimately led to the accelerated FDA approval of pembrolizumab as an option for partial cases in CRC treatment (95). In 2012, 18 out of 207 colorectal cancer patients participated in a multicenter clinical phase I trial of PD-L1 monoclonal antibody. However, no objective reflection (full or partial remission) was detected (96). Although PD-L1 monoclonal antibodies are successful in preventing some solid tumors, they are not very effective in treating colorectal cancer. Later, J. Bendell, J. performed atezolizumab, bevacizumab in combination with FOLFOX, and the combination of MEK inhibitor cobimetinib with atezolizumab in CRC patients, demonstrating improved efficacy, enhanced CD8T cell infiltration, and MHC I expression. Therefore, the PD-L1 therapeutic alliance for CRC has a promising future. Early trial results of PD-1 monoclonal antibodies appear promising, particularly in colorectal cancer patients with the dMMR staging. PD-L1 inhibitors are less active as monotherapy but have enhanced efficacy in combination, potentially extending the indications for ICI to patients with the pMMR staging (53, 97). After the discovery and clinical application of the anticancer capabilities of ICIs, the high occurrence of drug resistance (both primary and acquired) has emerged as a critical concern in the area, limiting their clinical applicability.

### Immune escape—the key to drug resistance

ICIs have become a crucial part of the treatment of colorectal cancer. However, not everyone can benefit from it, nor does always benefit. According to R. Cohen et al., five out of 38 mCRC patients treated with ICIs exhibited primary resistance, of which three were dMMR (98). The FDA-approved ORR for mCRC with nivolumab monotherapy is 31%, whereas nivolumab plus ipilimumab investigators evaluated an ORR of 55% (99). These characteristics imply the occurrence of dMMR CRC patients with intrinsic or emerging resistance to immune checkpoint drugs. Two types of resistance to ICIs can be roughly categorized: (1) Primary resistance, which generally refers to patients who do not respond to ICIs at all from the start and progress quickly or eventually. (2) Acquired resistance, which refers to patients who respond to ICI therapy initially but then progress clinically and/or radiologically (100). The current technique for overcoming primary resistance is to employ combination therapy that mixes immunosuppressive medicines with additional biologics, such as PD-1 inhibitors in combination with tumor vaccines (NCT03289962). In contrast, the mechanism of acquired drug resistance is more complex and has not been studied in detail with precision. Different drug-resistant populations develop resistance at different rates and to varying degrees, but there is no fundamental difference between them. In a nutshell, it is a tumor immune escape mechanism.

#### Medication resistance major issue—Immune escape hypotheses

In 1909, Paul Ehrlich made the initial discovery and suggestion that tumor formation was caused by an immune system dysfunction and that the immune system itself might limit tumor development through investigations into transplantable breast tumors in mice. At the time, this was not universally accepted by the academic world (101). In the middle of the 20th century, fifty years after Ehrlich’s theory, Frank Macfarlane Burnet and Lewis Thomas proposed that mutations in somatic cells were inevitable in the human body but that the body’s internal homeostasis could be eliminated by a substance or mechanism in the immune system that could eliminate potentially dangerous mutant somatic cells (102). In the 21st century, Grulich et al. observed a significant incremental increase in cancer risk and a similar pattern in both population groups through a cohort study of AIDS patients and transplant immunosuppressed individuals, indicating a correlation between cancer incidence and immunodeficiency (103). Eventually, Gavin P. Dunn and Robert D. Schreiber proposed a more systematic and comprehensive theory of Cancer Immunoediting to characterize the immune system’s defense of the host and its influences on the alteration of tumor disease, ultimately leading to the concept of immunological escape. Three processes are involved in cancer immunoediting: elimination, equilibrium, and evasion. Effective immune evasion will result in the mutation of tumor cells that are insensitive to immunological monitoring, their elimination in the form of genetic or epigenetic alterations, and the initiation of uncontrolled growth that leads to clinically diagnosed cancer (104).

#### MSI-related immune escape

**Figure 3:**
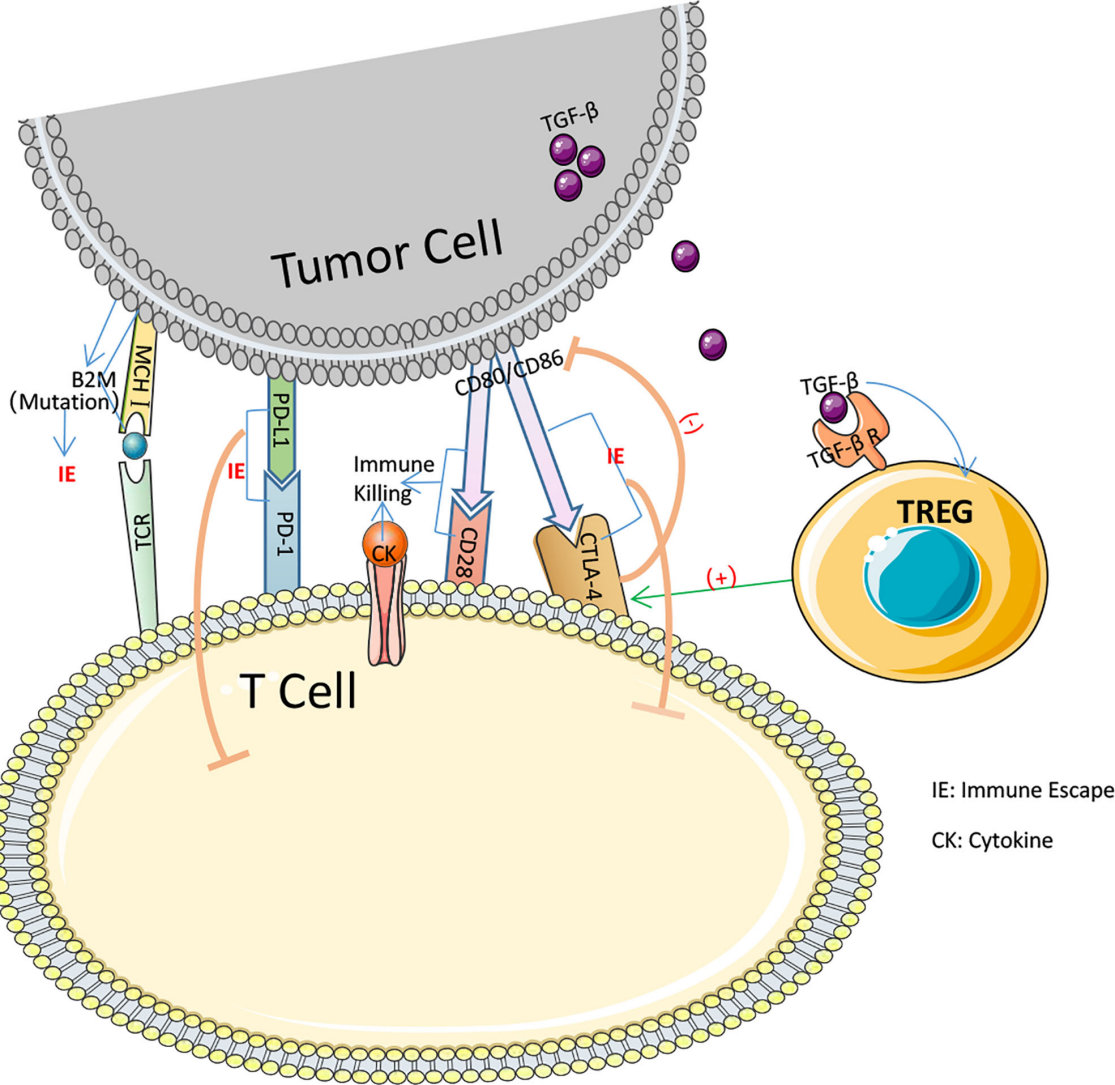
Several pathways by which immune escape occurs in colorectal cancer.

pMMR is frequently associated with primary immunological resistance, whereas ICIs is more frequently used in dMMR. In 48 percent of all trials, significant tumor shrinkage or no advancement was reported after PD-1 blocking therapy, while in 52 percent of all patients, prompt tumor enlargement after therapy or shrinkage followed by enlargement was observed (105). In MSI-H CRC treatment, ICIs resistance may result from many MSI-H related immune escape symptoms. Insertion or deletion of nucleotides in microsatellite mutations results in translational frameshifts that affect the translational expression of proteins that may express MSI-specific shift peptides (FSP), eliciting intense local and systemic anti-tumor immune responses in the host while evading immune control through various mechanisms (106–109). Nicolas J. Llosa et al. discovered that MSI-H CRC had a high degree of Th1 and CTL activation in the microenvironment (110). Regrettably, MSI-H CRC progression remained brisk. All of this demonstrates that MSI-H has a specific immune escape that contributes to the progression of CRC development (**Figure 3**). Following immunological monitoring of MSI-H, it was realized that, probably during tumorigenesis, MSI-H colorectal cancer cells chose tumor cells with a defective antigen handling mechanism (APM) to promote their proliferation. Matthias Kloor et al. evaluated the expression of Human Leukocyte Antigen Class I Antigen (HLA-I) subunits in 20 MSI-H CRC and 20 MSS CRC tissues using monoclonal antibodies specific to APM components. Total HLA-I antigen loss was observed in 12 of 20 MSI-H lesions (60%) but in just 6 of 20 MSS colorectal lesions (only 30%). In other words, the MSI-H phenotype of colorectal cancer was associated with a high prevalence of deficient HLA-I antigen presentation (111). HLA-I antigens transport polypeptides from cells to the immune system. When a tumor-specific antigen is present on the cell surface, CD8+ T lymphocytes are able to recognize the antigen and then secrete cytotoxic substances to induce antitumor immunity. Thus, diminished HLA-I antigen presentation is an effective defense against the cytotoxic T cell onslaught (112, 113). The HLA-I complexus is composed of the HLA-I heavy chain, B2M, and a peptide fragment including the molecular chaperone Tapasin, Calnexin, Calreticrin, and ERp57.39, which are assembled in the endoplasmic reticulum in a progressive manner. 14 of 124 CRCs (11%) examined by C.M. Cabrera et al. IHC and Mab analysis exhibited complete deletion of HLA-I. Four cases exhibited inactivation of the 2M biallelic sites and accumulation of intracytoplasmic HLA-I heavy chains, which may result in a failure of T cell identification in the immunological response, as determined by simultaneous MSI-H and RT-PCR analysis (114). 45 β2M mutations have occurred at an advanced stage of carcinogenesis in MSI-H CRC (115). Truncating mutations in the β2M gene, which is involved in the folding and transport of MHC I molecules, can affect the expression of MHC I on the APC surface, leading to reduced antigen presentation and immunotherapeutic resistance. It is believed that abnormal mutations in β2M constitute a key mechanism of tumor resistance to T cell-mediated immune responses and a source of immunotherapeutic resistance (116, 117). Changes to a decrease in β2M and HLA-I may present an opportunity to reverse immunological resistance.

#### TMEs develop ICIs resistance as a result of immune evasion

The dynamic tumor microenvironment (TME) may be closely related to the mechanism of drug resistance development. TME encompasses both anti-tumor immune and pro-tumor growth cells, and the intricate interplay between anti-tumor immunity and immunosuppression alters the balance between tumor growth and tumor elimination on a continuous basis. With the advancement of research and technology, the significance of the relationship between cancer and the immune system is becoming recognized, and in 2011, evading immune destruction was identified as one of the defining characteristics of cancer (118). In addition to cancer cells, the tumor microenvironment now comprises a heterogeneous population of immune cells, interstitial cells, endothelial cells, cancer-associated fibroblasts, and their related secreted factors (119). TME is also a key immune escape and cancer proliferation stimulator (120). Crucial to carcinogenesis is the interaction of malignant cells with diverse cells inside the TME. The TME contains certain immune cells, including T and B lymphocytes, tumor-associated macrophages (TAM), dendritic cells (DC), natural killer (NK) cells, neutrophils, and myeloid-derived suppressor cells (MDSC); also contains stromal cells (such as cancer-associated fibroblasts (CAF), pericytes and mesenchymal stromal cells); the extracellular matrix (ECM) and other secreted molecules like growth factors, cytokines, chemokines and extracellular vesicles (EV); and the network of blood and lymphatic vessels that are co-connected and not only influence each other but are also associated closely with tumor cells (121–124). As described in the theory of cancer immune editing, cancer cells are inactivated by anti-tumor immunity in the tumor microenvironment in the early stages of cancer development, but as the tumor proceeds, the tumor stalemates with the immune system and eventually the clinical manifestations of the tumor cells must undergo immune escape, a process in which TME is accompanied by significant disruption of the cellular immune response. The work of Joel Crespo et al. demonstrates that in the case of late immunosuppressive TME, TIL activation and functional expression are restricted, T cell depletion increases, tumor cells continue to grow meanwhile tumor cells leaving the TME are attacked by other immune cells, thus inferring the existence of immune escape under certain conditions in TME (125).

Continuous angiogenesis, one of the features of tumor development, plays a driving role in the process of TME shift in the direction of immune escape occurrence (118). Rapid tumor proliferation is always accompanied by angiogenesis to meet the needs of tumor cells for oxygen and nutrition (126). Additionally, tumor cells’ aberrant angiogenesis is unable to carry enough oxygen. Reduced oxygen levels are present in 50%–60% of solid tumors (127). During the course of the tumor, the high glycolysis rate of the tumor cells generates a significant amount of acidic chemicals, which causes the TME’s weak acidic environment to stand out (128). While this is happening, the structural and functional abnormalities of the tumor’s vasculature cause local blood leakage, which raises interstitial fluid pressure (IFP). High IFP then makes it harder for tumor tissue to be perfused, which worsens the tumor’s hypoxic, acidic, and high IFP microenvironment (129, 130). In these circumstances, TME stimulates the production of chemokines to encourage the infiltration of immunosuppressive cells, which TME tilts toward immunosuppression (131). Additionally, the hypoxic environment might prevent effector T cells from penetrating. Vascular endothelial growth factor lowers T-cell adhesion molecule expression. As well as causing endothelial cells to express Fas ligands through the Fas/FasL signaling pathway, VEGF-A, IL-10, and prostaglandin E2 (PGE2) decrease T cell mobilization and invasion by killing CD8+ T cells and endothelial cells (132). In addition to significantly reducing the recruitment of immune-suppressive cells to the tumor, blocking intracellular angiogenesis in tumor cells also promotes the infiltration of effector T cells (133). Bevacizumab, as a VEGF monoclonal antibody, received FDA approval in 2004 for the treatment of CRC (134).

From the development of anti-tumor immunity to immune escape, the changes in the TME deserve our research attention. In CRC, there are also dynamic changes in the TME, the mechanisms of which include altered antigenicity of tumor cells and the consequent production of a range of immunosuppressive mediators that modify the interactions between cells in the TME (135). Inhibits the functions that ICIs are supposed to perform.

## Conclusion

This review focuses on immunotherapy and immune escape related drug resistance reverse in colorectal cancer, which is an important and rapidly expanding field. Colorectal cancer has long afflicted patients with the danger and uniqueness of being a built-in organ cancer that is not easily identified and treated at an early stage, and at an advanced stage, has a large risk of spreading and is difficult to cure. Colorectal cancer, as a leading cause of death worldwide, will account for approximately 3% of all deaths in 2020, with the incidence rate increasing year by year (1). Traditional cancer treatments, including surgery, chemotherapy, and radiation therapy, have limitations and cannot eradicate the tumor entirely. Moreover, surgery will alter the function of patients’ organs; chemotherapy will exacerbate anemia and weakness, and long-term chemotherapy resistance is unavoidable; radiotherapy is radioactive, and white blood cell depletion, hair loss, and even systemic reactions such as radioactive stomatitis and radioactive esophagitis may occur. Immunotherapy has emerged and advanced as a result of the discomfort and side effects during treatment and the bad prognosis following treatment.

More and more relevant clinical studies have been conducted with the debut of ICIs in immunotherapy and the FDA’s approval of ICIs as CRC treatment agents. However, we should highlight that not all clinical studies are planned to include a discussion of genotyping concerning ICIs. Perhaps adding more genotyping requirements at enrollment and researching more precise dMMR/pMMR categorization will help us conduct clinical trials more correctly and expand applications with the introduction of ICIs. In addition to the fact that we could not uncover accurate specific biomarkers for CRC, particularly in MSS/pMMR CRC, how to overcome the barriers to make ICIs effective is critical to the success of ICIs in CRC (136, 137). Currently, it is understood to be successful to combine immune checkpoint inhibitors with chemotherapeutic methods (138, 139). 5-Fluorouracil (5-FU) was the first chemotherapeutic drug for CRC that was proven to be successful. In research by Javadrashid et al. (140), it was discovered that 5-FU therapy decreased pancreatic cancer cells’ expression of tumor PD-L1. The findings of Afshin Derakhshani et al. showed that capecitabine, a medication that acts as a precursor to 5-FU, significantly reduced CTLA-4 in CRC tumor cells (141). But Van Der Kraak et al. did show that 5-FU therapy led to PD-L1 upregulation in CRC cells (142). How 5-FU functions *in vivo* results in conflicting scenarios with two traditional IC mechanisms. In order to meet our therapeutic goals and increase patient survival rates, more consideration should be given to how to combine the medications to concurrently inhibit PD-L1 and CTLA-4 expression. In the future, we may need to think more about and do more research to see whether combination treatments can reduce the occurrence of immunological resistance and which medications can be used in conjunction with ICIs to provide greater therapeutic results. TME is a similar dynamic *in vivo* mechanism, analogous to the dynamic changes in drug resistance. The pursuit of the potential to reverse drug resistance in TME appears promising. In comparison to typical immune cells, the role of immunosuppressive cells in medication resistance cannot be overlooked. It has been demonstrated that blocking immunosuppressive cells improves the efficacy of ICIs. In addition, specific indicators for determining the success of immunotherapy in patients with colorectal cancer are still unknown. To minimize harmful side effects and maximize the therapeutic efficacy of immunotherapy, particular indices will be selected. In particular, it provides more reliable clinical treatment guidelines for the monitoring of immune-related adverse events (IrAEs).

In future research, it will be essential to comprehend the precise mechanisms and toxicity measurements by which ICIs build resistance. This will promote the development of new diagnostic and therapeutic options to address the limits of the present treatment for ICIs and assist a greater number of CRC patients.

## Author contributions

All authors planned and wrote the manuscript and contributed to the article and approved of the submitted version.

## Funding

The present study was supported by the National Natural Science Foundation of China (grant no. 81872264).

## Acknowledgments

Drawing some of the figures included utilizing images from Servier Medical Art. A Creative Commons Attribution 3.0 Unported License is used to license Servier Medical Art (https://creativecommons.org/licenses/by/3.0/).

## Conflict of interest

The authors declare that the research was conducted in the absence of any commercial or financial relationships that could be construed as a potential conflict of interest.

## Publisher’s note

All claims expressed in this article are solely those of the authors and do not necessarily represent those of their affiliated organizations, or those of the publisher, the editors and the reviewers. Any product that may be evaluated in this article, or claim that may be made by its manufacturer, is not guaranteed or endorsed by the publisher.
